# Effect of age, sex, physical activity and meteorological factors on haematological parameters of donkeys (*Equus asinus*)

**DOI:** 10.1007/s00580-014-2026-3

**Published:** 2014-10-30

**Authors:** Friday Ocheja Zakari, Joseph Olusegun Ayo, Peter Ibrahim Rekwot, Mohammed Umar Kawu

**Affiliations:** 1 Department of Physiology, Faculty of Veterinary Medicine, Ahmadu Bello University, PMB 1045, Zaria, Nigeria; 2 National Animal Production Research Institute (NAPRI), Ahmadu Bello University, PMB 1096, Zaria, Nigeria

**Keywords:** Age, Sex, Physical activity, Haematological parameters, Donkeys

## Abstract

The review examines the role of blood in homeostasis, diagnosis and treatment of disease as influenced by meteorological stress factors, age, sex and physical activity of the donkeys. Haematological parameters play a crucial role in clinical diagnosis of infectious and parasitic diseases, in assessing the responses of donkeys to treatment and in prevention of diseases. The changes in blood values are important in evaluating the responses of the animals to various physiologic conditions. In conclusion, haematological values of donkeys are largely influenced by age, sex, physical factors of the environment and physical activity, and consideration of the factors will aid accurate diagnosis and therapeutic evaluation of equine diseases.

## Introduction

The donkey (*Equus asinus*) has been used as a working animal for at least 5000 years mainly for transportation, conveying farm produce to the market or pulling of carts and also for farm tillage (Starkey and Starkey 2004). Presently, donkeys are used in the production of milk for children who are allergic to bovine milk (Carrocio et al. 2000; Muraro et al. 2002; Caldin et al. 2005; Mansueto et al. 2013) and in animal-assisted therapy and activity in humans (Borioni et al. 2012). The population of donkeys is on the increase in Africa, and the animals are increasingly becoming important in transportation of farm produce. In Nigeria, donkeys are used mainly in transportation of goods and agricultural produce (Blench 2004).

Haematological examination has been performed for a variety of reasons: as a screening procedure to examine general health of the animal, as an adjunct to a patient’s infection and to evaluate the progress of certain disease conditions. Haematological values play a vital role in clinical diagnosis, the assessment of patient before surgical procedure and in monitoring the progress of animals to treatment (Mori et al. 2004; Satue et al. 2009; Yaqub et al. 2013). The diagnosis and treatment of diseases in animals greatly depends on clinical examination and haematologic results, which reflect biological variation. This in turn requires the understanding of haematological profiles of an, apparently, healthy animal, whose physiological parameters provide invaluable information regarding the health status of the animal (Kral and Suchy 2000; Mori et al. 2003, 2004; Cetin et al. 2009). Inspite of the clinical significance, correct haematological interpretation and analysis are sometimes difficult because many factors considerably influence the haematological parameters in goats, pigs, rabbits, camels, cattle, horses and donkeys, including age (McFarlane et al. 2001; Caldin et al. 2005; Gurgoze and Icen 2010; Melo et al. 2013; Padalino et al. 2014; Girardi et al. 2014;), sex (Cetin et al. 2009; Mirzadeh et al. 2010; Uluisik et al. 2013; Girardi et al. 2013, 2014), breed (Tibbo et al. 2004; Lacerda et al. 2006; Orozco et al. 2007; Šimpraga et al. 2013), seasons (Dmoch et al. 2008; Adenkola et al. 2011; Mirzadeh et al. 2010; Abdelatif and Alameen 2012; Babeker et al. 2013; Šimpraga et al. 2013), diet (Ekenyem and Madubruke 2000; Iyayi 2001; Kumar et al. 2005), health status and sub-clinical disease (Rose and Hodgson 1994; Petrov et al. 2011; Kalai et al. 2012) work or exercise (Piccione et al. 2001; Zobba et al. 2011; Kedzierski et al. 2009; Miranda et al. 2011) and pregnancy (Harvey et al. 1994; Orozco et al. 2007; Satué et al. 2010a, b; Farooq et al. 2011; Okonkwo et al. 2011; Aoki and Ishii 2012).

The aim of the present review was to determine the effect of age, sex, physical factors of the environment and physical activity on haematological parameters of donkeys.

## Domestication and uses of donkeys

Domestication of the donkey transformed ancient societies and initiated the development of nomadic pastoral communities in Africa and the expansion of land-based routes in the Middle East (Marshall 2007; Rossel et al. 2008). Marshall (2007) stressed on the importance of African pastoralist use of donkeys for transport during long-distance movements, for women’s daily tasks, such as fetching water or firewood, and in the sustainable management of rangelands. The relationship between humans and the ancestors of modern donkeys was established by pastoralists, who had to use the animals to assist them to survive the harsh Saharan terrain in Northern Africa more than 5000 years ago (Kimura et al. 2011). Today, donkeys play critical role in assisting African herders to cope with unpredictable climatic fluctuations. They provide greater mobility with which to face erratic rainfalls and are used for carrying loads including firewood, water, household structures, goods and children (Marshall and Weissbrod 2011). In India, donkeys have been used to carry arms and ammunition to the difficult terrains that mechanized vehicles cannot reach easily (Singh et al. 2005). The ultimate use of donkeys for human therapeutic purpose is in the field of onotherapy, a method frequently used for people who have disabilities and discomforts (Karatosidi et al. 2013).

In ancient civilization, donkeys were used for different purposes than today. They were not only used as pack (load-carrying) animals but their milk, skin and meat were useful. Donkey milk has recently generated scientific interest because of its attractive nutrient and functional components. It is considered a valid substitute for infants with severe IgE-mediated cow’s milk protein allergy (Monti et al. 2007). Donkey milk is a potent vasodilator, making it potentially useful in the prevention of atherosclerosis (Tafaro et al. 2007) and has been shown to exert suppressive effect against human lung tumour in vitro (Mao et al. 2009). The milk was highly prized because it contains more sugar and protein than bovine milk. It is also a good substitute for babies and the sick (Criscione et al. 2009). Donkeys have recently gained new application with the use of their milk to feed humans with allergic disorders. The Ragusana and Martina Franca breed from Sicily, Italy, is used to produce milk for human consumption (Caldin et al. 2005).

## Homeostatic role of blood

Blood is a special type of regulatory, protective and homeostatic connective tissue, comprising formed elements (erythrocytes, leucocytes and platelets) and plasma (Nasyrova et al. 2006; Eze et al. 2010). The main function of the blood is the transportation of oxygen, which is achieved through the presence of conjugated metallo-protein known as haemoglobin (Ashton 2013). Blood values are used for evaluating stress and the well-being of the animal. Its profiles in animals provide means of assessing the internal environment and understanding the causes of impairment in homeostasis as evidenced by marked fluctuations in physiogical indices under different internal and external environmental conditions (Sattar and Mirza 2009). Blood constituents reflect the influence of normal physiological and pathological states of the animals, including thermal stress (Satue et al. 2009). For example, packed cell volume (PCV) and haemoglobin concentration (Hb) are used in the assessment of metabolic profile of animals (Kida 2002). Neutrophil blood counts are the consequence of a very dynamic feedback mechanism, which are controlled by different environmental and genetic factors that remain largely unknown (Von Vietinghoff and Ley 2008). At present, neutrophil/lymphocyte ratio (N:L) is a well-accepted indicator for stress assessment in animals (Rajion et al. 2001; Goundasheva et al. 2005; Stanger et al. 2005; Quiñonero et al. 2009; Holub et al. 2012).

Platelets are important in physiologic and pathologic processes of inflammation, tumour metastasis, haemostasis, wound healing and host defense (Semple et al. 2011). Variation in haematological parameters is a reflection of an animal’s response to its external and internal environments (Esonu et al. 2001).

## The use of haematological parameters in diagnosis and treatment

Inappropriate reference values for haematology increase the risks of unnecessary investigations and diagnostic failures. Species, breed, environment, handling and physiological state influence haematologic and biochemical variables (Šimpraga et al. 2013; Etim et al. 2013; Girardi et al. 2014). Haematology and serum biochemistry are important diagnostic aids use to assess metabolic status in starved equids and to rule out pathologic conditions. The knowledge of the haematologic findings in emaciated equine is useful in scoring the intensity of emaciation, in establishing a prognosis and a plan to recover the health status of these animals (Muñoz et al. 2010). Haematological values during different physiological situations are beneficial in ensuring accurate diagnosis of various pathological and metabolic disorders, which may adversely affect livestock performance (Sattar and Mirza 2009). The comparison between individuals using reference data is necessary, based on normal variations due to sex, age and breed in order to increase diagnostic precision in clinical situations (Satue et al. 2009).

## Haematological responses to stress factors

The haematological profile is important in the determination of physiological changes occurring in animals (Kumar and Pachaura 2000; Anderson et al. 1999). Haematological parameters such as packed cell volume (PCV), total erythrocyte count (RBC), mean corpuscular haemoglobin (MCV), mean corpuscular haemoglobin concentration (MCHC), mean corpuscular haemoglobin (MCH) and total differential leucocyte counts (WBC) are used to evaluate adaptability of animals to adverse environmental conditions (Koubkova et al. 2002). Research in haematology is of ecological and physiological importance, and the findings are of value in understanding the relationship between blood characteristics and the environment (Ovuru and Ekweozor 2004) and selection of animals with superior genetic traits (Mmereole 2008; Isaac et al. 2013). Strenuous exercise or work results in severe haematological, metabolic, acid-base and ionic changes in animals, which are specie-specific, and are influenced by the magnitude of the cumulative effects of physiological and physical trauma, related to the stressful events (Hassanein 2010). Acidosis and physical stress result in disturbances in haematological parameters such as haemoglobin and haematocrit levels (Moyes et al. 2006).

Erythropoietic responses observed during exercise are the results of myriads of interacting mechanisms that involve oxidative stress and haematopoietic system (Lorena et al. 2006). Changes in the osmotic fragility of equine erythrocytes during exercise are important indicator of stress and intravascular haemolysis (Hanzawa and Watanabe 2000). Cuniberti et al. (2012) observed that exercise results in significant increase in erythrocyte count, haemoglobin concentration, PCV and neutrophil, lymphocyte and monocyte counts. A direct relationship exists between leucocyte profiles, stress and glucorticoid levels (Vleck et al. 2000). Glucorticoids secreted in stress act to increase the number and percentage of neutrophils while decreasing the number and percentage of lymphocytes (Johnstone et al. 2012). Thus, neutrophil:lymphocyte ratio is of diagnostic value in stress (Altan et al. 2003; Walsh et al. 2005; Zazula et al. 2008).

## Effect of age on haematological parameters

Effects of age on haematological parameters have been documented in various breeds of horses (Hernández et al. 2008; Satue et al. 2009). Grondin and Dewitt (2010) and Sgorbini et al. (2013) reported that newborn foals have high PCV, RBC and Hb because erythrocytes are of foetal origin. Thus, the involvement of the liver and spleen in erythropoiesis and probably due to the transfusion of placental blood to the foal at birth may result in increased erythrocytic values. The high values of PCV, haemoglobin concentration and erythrocyte count observed in foals may also occur as a result of functional changes in haematopoiesis and high water content in foals or due to haemoconcentration because foals depend mainly on milk. Sgorbini et al. (2013), Muñoz et al. (2012) and Grondin and Dewitt (2010) observed that PCV, Hb and erythrocyte count decreased significantly after the first 48 h of life, probably as a result of haemodilution, physiological destruction of erythrocytes by the spleen and decreased erythropoietin production secondary to increased blood oxygenation by lungs after birth. Muñoz et al. (2012) also reported higher PCV, RBC and Hb in Spanish foals than those in adult Spanish horses. There is an increase in erythrocyte counts, PCV and Hb in donkeys as they get older (Etana et al. 2011). A reduction of erythrocyte count with a compensatory increase in MCV and MCH in older Carthusian pregnant mares and Spanish purebred mares have been reported (Hernández et al. 2008). Satue et al. (2009), observed increased MCH, MCV and MCHC in adult horses. In a study conducted on working donkeys, the RBC, Hb, MCV, MCHV, PCV and MCH are higher in adult donkeys than in young ones (Etana et al. 2011). According to Folch et al. (1997), young donkeys had significantly lower values of MCV and MCH as well as higher counts of leucocytes, segmented neutrophils, immature neutrophils and eosinophils than older animals. PCV and Hb increase with age in working horses (Pritchard et al. 2009). MCV in foals less than 1 year is significantly lower than that of the adult horses. Lippizan foals aged between 4 months and below have higher erythrocyte count and PCV than stallions and mares of the same breed (Cebulj-Kadunc et al. 2002). Brommer et al. (2001) reported an increase in neutrophil and lymphocyte counts during the first months of life. Uluisik et al. (2013) observed significant difference in RBC, PCV and MCV in horses of different ages. Adult working donkeys have higher PCV, RBC, Hb, MCV, MCHC and MCH than young donkeys (Lemma and Moges 2009). Old pregnant Andalusian mares have lower total leucocyte and lymphocyte counts, compared to younger ones (Satue et al. 2009). In addition, higher leucocyte count in younger animals when compared to adult was observed in Kathiawari horses in India (Gupta et al. 2002). The decrease in leucocyte count in healthy, aged horses may be related to age-associated decrease in immunocompetency (Mcfarlane et al. 2001).

## The role of sex on haematology of donkeys

The sex of an animal affects blood parameters in many animal species, and values in females are lower than in males. The PCV in male Red Sokoto goats are higher than in females. Slight differences or variation in blood parameters have been reported in equids. Male horses have slight RBC, Hb and PCV than female. Mares have been reported to have higher MCH values (Hernández et al. 2008; Satue et al. 2009). Significant differences related to sex of thoroughbred foals were reported with regard to PCV at 1 year and WBC at day one (Uluisik et al. 2013). Sex variation in immune function is well established in vertebrates. Total leucocyte count is higher in mares than stallions of the Spanish purebred (Hernández et al. 2008). However in other studies, no significant differences between male and female horses were obtained (Lacerda et al. 2006). The increase in haematological parameters in males compared to females may be due to the effect of androgens, which stimulate erythropoiesis (Kelani and Durotoye 2002). In a study conducted to determine baseline values for haematology in working donkeys (Etana et al. 2011), there were significant variations in total leucocyte, MCV and neutrophil counts between sexes. Male Przewalski horses were characterized by significantly higher erythrocyte count and immature neutrophilic granulocytes than female Przewalski horses (Tomenenendalova et al. 2014). In the experiment conducted by Tomenenendalova et al. (2014), stallions were also observed to have a relatively higher haemoglobin concentration and leucocyte and monocyte counts than mares but without significant significance. The PCV and haemoglobin concentration are lower in mares than in working horses with low body condition scores (Pritchard et al. 2009). The PCV in female foals was reported to be higher than in colts of 1–2 months of age. The work of Mori et al. (2004) and Gul et al. (2007) on donkeys reported no sex differences in haematological parameters. There were variations in RBC, MCV and neutrophil counts between different male and female donkeys (Etana et al. 2011). Leucocyte count was higher in female Spanish purebred horses than males (Hernández et al. 2008). Studies conducted by Hernández et al. (2008) and Satue et al. (2009) showed that male horses have slightly higher PCV, RBC and Hb than female horses. The slight increase in erythrocytic parameters in males may be attributed to the effect of androgens, which stimulate erythropoiesis (Gaspar-López et al. 2010). Mikniene et al. (2014) established that female Zematukai horses have higher Hb, PCV, RBC and MCHC than male horses. Zinkl et al. (1990) observed that female donkeys had higher values of MCHC and leucocyte and neutrophil counts than adult males.

## Influence of physical factors of the environment on haematological parameters

Season and meteorological parameters are external factors that control the dynamics of blood constituents in the equine (Satué et al. 2011). In the equine, seasonal changes may decrease some haematological parameters, including RBC, Hb and PCV. Season is an external factor that controls the dynamics of blood constituents in equines (Satué 2004; Satué et al. 2011). In Carthusian mares, PCV, RBC and MCV are higher in the summer than any other seasons (Satué et al. 2011). It has been suggested that extreme cold reduces the number of erythrocytes due to the reduction in the half-life of the erythrocytes (Ruiz et al. 2004). Seasonal changes in ambient temperature, relative humidity and air velocity influence the physiological responses of animals. Changes in haematological values such as PCV, RBC, MCV and MCHC are used in determining the adaptation of animals to the environment (Koubkova et al. 2002). In Polish warmblood mares, significant increases in neutrophil count, MCH and MCHC were obtained in the summer than in the winter and spring (Dmoch et al. 2008). Blood parameters in Arabian horses such as PCV, Hb and RBC are significantly lower in the summer, compared with the winter season (Gill et al. 1979). Gill and Kompanowska-Jezierska (1986) reported that Arabian mare and thoroughbred horses have higher PCV, RBC and Hb during the winter than the other seasons. In the tropics, animals are predominantly kept outdoor, and they are exposed to direct solar radiation during the day and other thermal stressors, including high ambient temperature, high humidity and rainfall, exerting significant effect on their performance (Silanikove 2000; Scharf 2008; Hetem et al. 2011). Animals under heat stress must alter their behaviour and physiology to increase heat loss, reduce heat production and restore homeostasis. This is done at the expense of productivity (Huynh et al. 2005; Lin et al. 2009). Ungulates in the arid regions are able to survive extreme environmental conditions due to behavioural and physiological adaptations of the animal to combat these adverse conditions (Cain et al. 2006; Sneddon et al. 2006). The environment in which equines are raised influences the ability of the animals to maintain thermal equilibrium, which is, in turn, related to their thermal characteristics and regulatory physiological mechanisms (Castanheira et al. 2010). In the Northern Guinea Savanna zone of Nigeria, the early rainy season was observed to be stressful to packing donkeys (Ayo et al. 2008). Heat stress causes cascade of changes in the physiological mechanisms of animals. Increase in physiological parameters of donkeys above the normal range indicates that the animals are stressed (Minka and Ayo 2007). Extreme hot and cold ambient temperatures affect animals, which is evident in fluctuations of physiological responses to combat the thermal environmental stress (Pandey et al. 2012).

## The relationship between physical activity and haematological parameters of donkeys

Physical activity or work in animal is a commonly recognized stress factor which can influence haematological parameters. Physiological changes in haematological parameters occur in response to exercise and training in thoroughbreds and standardbreds (Fazio et al. 2011). The rate of metabolism of neutrophils is higher in regularly trained horses than in animals that engage in mild physical activity (Krumrych 2009). Piccione et al. (2008) observed that platelet aggregation in exercising horses is different from that in resting horses. Physical activity or exercise exerts effect on the daily rhythm of platelets in horses. Lemma and Moges (2009) in Ethiopia established that clinical and haematological values were not affected by the degree of work performance in working donkeys. However, serum biochemical values, except total protein, were significantly affected by workload. According to Olaifa et al. (2012), packing significantly decreases the PCV and RBC of donkeys, while the neutrophil/lymphocyte ratio rose significantly after the donkeys were subjected to packing. Oxygen consumption and average heart rate in donkeys and running at a maximum speed significantly increased heart rate in animals, compared to values obtained in non-exercising horses (Mueller et al. 1994). Changes occurring in haematologic parameters induced by physical work have been well documented (Hinchcliff et al. 2002; Lorena et al. 2006).

Erythrocyte osmotic fragility is used to determine the level of stress in animals (Hesta et al. 2008). Alterations in the erythrocyte osmotic fragility occurs during strenuous work in horses (Hanzawa and Watanabe 2000). Metabolic responses to exercise differ between Andalusian horses and other breeds, although changes in activities of plasma muscle enzymes have not been reported, and most useful information is obtained from animals subjected to different training programmes (Muñoz et al. 2002). Rovira et al. (2007) observed that exercise competitions induce mild to moderate alterations in haematologic and biochemical parameters; consistent with splenic contraction, increased lipolysis and utilization of anaerobic pathways involved in energy re-synthesis in the muscle.

## Concluding remarks

Endogenous and exogenous factors such as age, sex and physical factors of the environment significantly affect or influence the haematological parameters of donkeys. Extreme meteorological factors, including high ambient temperature, relative humidity, solar radiation and wind velocity are stressors that alter the haematological parameters of donkeys. Frequent use of pack donkeys without adequate rest can lead to physical exhaustion due to work stress, thereby impairing haematological parameters of donkeys. Reference haematological parameters obtained from equids may enhance the current understanding of the haematologic values, which in turn may assist in equipping veterinarians with the ability to establish an accurate interpretation of laboratory data, critical in diagnosis, prognosis and treatment of diseases in donkeys.
